# Supplementary material for: The practice of blood cross-match request and transfusion in surgical patients at Debre Markos comprehensive Specialized Hospital, Debre Markos, Ethiopia, 2021/2022. A prospective study
Source: Ann Med Surg (Lond). 2022 Aug 8;80:104145. doi: 10.1016/j.amsu.2022.104145 (PMC9422072; doi:10.1016/j.amsu.2022.104145)
Supplement: Multimedia component 1 [file mmc1.docx]

| STROCSS 2021 guideline Check list |
| --- |

| Items name | Item descriptions |  |
| --- | --- | --- |
| Title | The Practice of Blood Cross-Match Request and Transfusion in Surgical Patients at Debre Markos Comprehensive Specialized Hospital, Debre Markos, Ethiopia, 2021/22. A Prospective Study | 1 |
| Abstract | **Background**: Blood transfusions play a great role to improve oxygen delivery to tissues for indicated surgical patients. Preoperative ordering of blood, especially in elective surgery, is often based on the worst-case assumptions, demanding large quantities of blood or overestimating the anticipated blood loss, of which little is ultimately used. This study aimed to assess the practices of blood requisite and transfusion in surgical patients.  **Method**: An institutional-based prospective study was conducted from September to February 2021/2022, in Debre Markos comprehensive Specialized Hospital. Socio-demographic data like age, sex, ASA status, type of anesthesia, and type of surgeries were taken preoperatively. The number of cross-matched and transfused data were collected from patient charts throughout the perioperative periods. Efficacy of blood utilization was evaluated using the following indices like cross-match to transfusion ratio (C/T ratio), transfusion probability (%T), and transfusion index (TI); a ratio of 2.5 and below, A value of 30% and above, and values of 0.5 or more respectively were considered indicative of significant blood usage and this study is registered with a research unique identifying number of [researchregistry7989](file:///C:\Users\user\AppData\Roaming\Microsoft\Word\Manuscript%20anals.docx) and reported in line with STROCSS 2021 guideline.  **Result**: In all procedures, among cross-matched blood units 64.1% were unutilized. Depending on the urgency of the procedures about 77.7% of cross-matched blood units were not utilized in elective patients. In emergency procedures, the majority (64.3%) of cross-matched units were transfused. The overall blood transfusion indices result was C/T ratio, %T, and TI was 2.9, 33.5%, and 0.7, respectively. The overall blood transfusion indices for elective and emergency surgery are set respectively as follows C/ T ratio (4.6 vs 1.5), %T (22 vs 78.8), and TI(0.4 vs 1.9). Among elective procedures, blood utilization was significant in orthopedic surgery with the value of C/T ratio 1.3, %T 66, and TI 1.4. In the rest of the elective procedures, blood transfusion indices were not significant.  **Conclusion**: The overall blood utilization indices in emergency surgical patients were better than in elective and preoperative grouping, screening, and hold (GSH) is sufficient for elective surgical procedures to decrease wastage of valuable supplies. | 2 |
| Background | Blood transfusions play a major role in the resuscitation and management of surgical patients to improve oxygen delivery to tissues (1). The preoperative ordering of blood, especially in elective surgery, is often based on the worst-case assumptions, demanding large quantities of blood or overestimating the anticipated blood loss, of which little is ultimately used (2-4).  In developing countries, no blood bank physician could determine the appropriateness of blood transfusion. Therefore blood is ordered by doctors and blood banks having no authority of overriding their requests has resulted in unnecessary ordering of blood products(5, 6). Preoperative over-ordering of blood for elective surgery has been documented in many studies (2, 3, 7, 8),that may results wastage of valuable supplies, biochemical reagents, burden to physical, and increases the cost of medical care (2, 4, 5, 7, 9-11).  There are variations in utilization of cross-matched blood across the world this may be due to arguments on the threshold level of hemoglobin below which a patient needs a blood transfusion, differences in surgical and anesthetic techniques, differences in case, preoperative anemia, and lack of availability of transfusion protocols(3, 10).  Due to lack of evidence-based guidelines for the transfusion of blood in most developing countries, ordering and transfusion of blood are frequently based on subjective decision-making skills and clinical knowledge (2, 7).  The use of blood conservation policies such as the Maximum surgical blood ordering schedule (MSBOS) can reduce unnecessary transfusion practices ,over-ordering of blood avoids unnecessary compatibility test,encourage proper returning of unused blood (3, 7),and MSBOS can save transfusion-related costs in the range of 23.3- 90.9 %(9, 12). | 3 |
| Objectives | General Objective  To assess blood cross-match requisite and transfusion practices in surgical patients at Debre Markos Comprehensive Specialized Hospital Debre Markos ,Ethiopia,from September to February 2021/2022.  **Specific objectives**  To assess the practice of blood cross-matching for elective and emergency surgical procedures.  To determine the maximum surgical blood ordering schedule (MSBOS) in elective and emergency surgical procedures. | 3&4 |
| **Methods** | **Materials and Methods**  An institutional-based prospective study was conducted from September to February 2021/2022, at Debre Markos Comprehensive Specialized Hospital. The hospital is found in East Gojam Zone located in northwest Ethiopia, and it gives General, Orthopedic,neurosurgery, Gynecologic, and Obstetric surgical services with both physicians and non-physician professionals. All patients who underwent elective and emergency surgery at the major operation room during the study period were included in the study. Patients who had incomplete or unclear documentation about blood order, cross-match, and transfusion were excluded from indices analysis and this study is registered with a research unique identifying number of [researchregistry7989](file:///C:\Users\user\AppData\Roaming\Microsoft\Word\Manuscript%20anals.docx) found with the link address [https://www.researchregistry.com/browse-the registry#home/?view_2_search=7989&view_2_page=1](https://www.researchregistry.com/browse-the%20registry#home/?view_2_search=7989&view_2_page=1) and reported in line with STROCSS guideline 2021(13).  In this study setting all clinicians used clinical signs and symptoms of anemia, their clinical judgment, and calculating maximum allowable blood loss during surgery to start blood transfusion rather than the numerical value of hemoglobin. The amount of blood loss during the intraoperative period was determined using abdominal packs, gauze swabs, a suction bottle, and the surrounding operation table.  **Data Collection**  Initially the data collectors gathering socio-demographic information like age, sex, and ASA status were recorded in the preoperative period while type of anesthesia and type of surgeries were taken during the intraoperatively and finally number of units cross-matched and transfused as well as the number of patients cross-matched and transfused were collected from patient charts during preoperative, intraoperative, and finally postoperative periods on the day of patient discharge. Additionally, blood transfusion-related data were cross checked in-hospital blood bank. Lastly the data were coded, entered, and analyzed using SPSS Version 26 and the efficacy of blood utilization was evaluated using the following indices (3, 7, 14-16).  (1) Cross-match to transfusion ratio (C/T ratio) = number of units cross-matched/number of units transfused. A ratio of 2.5 and below is considered indicative of significant blood usage.  (2) Transfusion probability (%T) = number of patients transfused/number of patients cross-matched × 100. A value of 30% and above was considered indicative of significant blood usage.  (3) Transfusion index (TI) = number of units transfused/ number of patients cross-matched. A value of 0.5 or more was considered indicative of significant blood utilization.  (4) Maximal Surgical Blood Order Schedule (MSBOS) = 1.5 × TI.  GSH (Group, Screen, and Hold) - Group: ABO and Rhesus grouping - Screen: Antibody screening only, no cross-match performance) - Hold: Keeping the blood sample and request form for 24 hours. (If no request for transfusion for 24 hours, the GSH will be expired automatically)(17, 18).  **Ethical Consideration**  Ethical clearance was obtained from the Research and Ethics Review Committee (RERC) of the school of medicine of Debre Markos University. Moreover, full clarification about the purpose of the study was made to the Authorized person of the health facilities. The purposes of the study were explained to the patient who was included in the study,additionaly written informed consent was obtained from each study subjects. | 5 |
| RESULTS | During the six months duration (September to February 2021/2022) 1008 elective and emergency surgeries were performed in Debre Markos Comprehensive Specialized Hospital. In this study the majority (78%) of all surgeries were ASA I ,and 65.3 %, 59.9% of patients were performed under elective bias and female study subjects respectively as shown in (**Table 1**).  Table 1: Socio-demographic and other characteristics of surgical patients in Debre Markos Comprehensive Specialized Hospital from September to February 2021/2022 (N=1008).   \| Variables \| \| N (%) \| \| --- \| --- \| --- \| \| Sex \| Female \| 604(59.9) \| \| Male \| 404(40.1) \| \| Age(year) \| < 18 \| 105(10.4) \| \| ≥18 \| 903(89.6) \| \| ASA status \| I \| 786(78) \| \| II \| 176(17.5) \| \| III \| 46(4.5) \| \| Type of anesthesia \| GA \| 685(67.9) \| \| RA \| 323(32.1) \| \| Urgency of surgery \| Elective \| 58(65.3) \| \| Emergency \| 350(34.7) \|   **Blood requisition and utilization**  The hospital blood bank was requested 850 blood units for 412 patients during the perioperative period. In all surgeries, 773 blood units were cross-matched out of the ordered units, and only 278(35.9%) units of blood out of cross-matched were transfused for 138 patients. This indicates that 64.1% of units of blood were unutilized. Even if the majority 65.6% of cross-matched units were done in elective procedures,around 77.7% of cross-matched blood were not utilized ,however in emergency procedures, the cross-matched better utilized. In this study 71 emergency surgical patients, 266 units of blood were cross-matched, out of which 171 (64.3%) units were transfused. The highest transfusion rate was conducted in uterine rupture and thoracic-abdominal trauma with 85.7% of cross-matched units transfused,but in appendectomy, ovarian cystectomy, cholecystectomy, and elective laparotomy none of the patients were transfused (**Table 2**).  Table2: Number of cross-matched and transfusion of surgical patients in Debre Markos Comprehensive Specialized Hospital from September to February 2021/2022 (N=1008).   \| Diagnosis(procedure) in OR schedule \| \| Number (%) \| Patients CM \| Patients T \| Unites CM \| Unites T \| \| --- \| --- \| --- \| --- \| --- \| --- \| --- \| \| Gynecologic and obstetric  procedures \| UVP (hysterectomy) \| 44(4.4) \| 44 \| 5 \| 44 \| 5 \| \| GTD(suction) \| 39(3.9) \| 30 \| 7 \| 35 \| 10 \| \| Ectopic pregnancy(emergency laparotomy) \| 18(1.7) \| 18 \| 9 \| 36 \| 16 \| \| Uterine rupture(emergency hysterectomy) \| 7(0.7) \| 7 \| 7 \| 35 \| 30 \| \| APH(emergency C/S) \| 18(1.7) \| 18 \| 12 \| 54 \| 32 \| \| Myoma (myomectomy) \| 40(4) \| 9 \| 2 \| 18 \| 3 \| \| Ovarian cyst (cystectomy) \| 18(1.7) \| 0 \| 0 \| 0 \| 0 \| \| Previous c/s scar(elective C/S) \| 115(11.4) \| 115 \| 25 \| 230 \| 41 \| \| Emergency C/S \| 93(9.3) \| 23 \| 15 \| 48 \| 22 \| \| General surgery \| BPH(open prostatectomy) \| 20(1.9) \| 20 \| 2 \| 45 \| 4 \| \| Thoraco -abdominal trauma (emergency Laparotomy) \| 11(1.2) \| 11 \| 8 \| 28 \| 24 \| \| Elective laparotomy \| 84(8.4) \| 5 \| 0 \| 5 \| 0 \| \| Subdural/epidural hematoma (drainage) \| 10(0.9) \| 10 \| 2 \| 15 \| 5 \| \| Goiter (thyroidectomy) \| 30(2.9) \| 30 \| 3 \| 42 \| 6 \| \| Breast Ca (Mastectomy) \| 30(2.9) \| 14 \| 3 \| 28 \| 5 \| \| Cholelithiasis (cholecystectomy) \| 60 (6) \| 0 \| 0 \| 0 \| 0 \| \| Appendicitis(appendectomy) \| 102 (10.2) \| 0 \| 0 \| 0 \| 0 \| \| Pediatrics surgery(elective and emergency) \| 97 (9.**7**) \| 6 \| 4 \| 6 \| 4 \| \| Others \| 96(9.6) \| 7 \| 2 \| 10 \| 2 \| \| Orthopedic procedures \| Elective \| 51(7.5) \| 24 \| 16 \| 44 \| 33 \| \| Emergency \| 25 \| 21 \| 18 \| 50 \| 42 \| \| Total \| \| 1008(100) \| 412 \| 138 \| 773 \| 278 \|   *Patients CM( number of patients cross-matched); Patients T(number of patients transfused); Unites CM(number of unites cross-matched); Unites T(number of units transfused); UVP(utro-vaginal prolapse); APH(Antepartum hemorrhage); GTD(gestational trophoblastic disease), OR(operation room); C/S(cesarean section); Ca(cancer); BPH(benign prostatic hyperplasia)  **Blood utilization indices of different procedures with respective MSBO**  In this study the overall blood transfusion indices result of C/T ratio, %T, and TI were 2.9, 33.5%, and 0.7, respectively. The overall blood transfusion indices for elective and emergency surgery are set respectively as follows C/ T ratio (4.6 vs 1.5), %T (22 vs 78.8), and TI(0.4 vs 1.9). But there are blood utilization indices variation among different types of surgical procedures. Among elective procedures, blood utilization was significant in orthopedic surgery with the value of C/T ratio 1.3, %T 66, and TI 1.4. In the rest of the elective procedures, blood transfusion indices were not significant as the C/T ratio, %T, and TI values mentioned in (**Table 3**). Among emergency procedures, blood utilization indices are not significant in epidural/subdural hematoma (bur hole). On the other hand, blood utilization is highest in uterine rupture (emergency hysterectomy) with the value of C/T ratio 1.2, %T 100, and TI 4.3 (**Table 3**).  Based on Mead’s criterion except for orthopedic procedures, the rest elective procedures didn’t need preoperative preparation of cross-matched blood and group, screen and hold (GSH) is enough. Orthopedic procedures, uterine rupture (emergency hysterectomy), Ectopic pregnancy (emergency laparotomy), APH (emergency C/S), Emergency C/S, and Thoraco -abdominal trauma procedures have significant utilization indices. Therefore, they need perioperative cross-matched blood preparation and perioperative transfusion (**Table 3**).  **Table 3:** Blood requisition and utilization in different procedures with respective MSBOS in Debre Markos Comprehensive Specialized Hospital from September, to February 2021/2022 (N=1008).   \| Diagnosis(procedure) in OR schedule \| \| Patients CM \| Patients T \| Unites CM \| Unites T \| C/T \| %T \| TI \| MSBOS \| \| --- \| --- \| --- \| --- \| --- \| --- \| --- \| --- \| --- \| --- \| \| Gynecologic and obstetric  Procedures \| UVP (elective hysterectomy) \| 44 \| 5 \| 44 \| 5 \| 8.8 \| 11.4 \| 0.1 \| GSH \| \| GTD(suction) \| 30 \| 7 \| 35 \| 10 \| 3.5 \| 23 \| 0.3 \| GSH \| \| Ectopic pregnancy(emergency laparotomy) \| 18 \| 9 \| 36 \| 16 \| 2.3 \| 50 \| 0.9 \| 1.3 \| \| Uterine rupture(emergency hysterectomy) \| 7 \| 7 \| 35 \| 30 \| 1.2 \| 100 \| 4.3 \| 6.4 \| \| APH(emergency C/S) \| 18 \| 12 \| 54 \| 32 \| 1.7 \| 66 \| 1.8 \| 2.7 \| \| Myoma (myomectomy) \| 9 \| 2 \| 18 \| 3 \| 6 \| 22 \| 0.3 \| GSH \| \| Ovarian cyst(cystectomy) \| 0 \| 0 \| 0 \| 0 \| NA \| NA \| NA \| GSH \| \| Previous c/s scar(elective C/S) \| 115 \| 25 \| 230 \| 41 \| 5.6 \| 21.7 \| 0.4 \| GSH \| \| Emergency C/S \| 23 \| 15 \| 48 \| 22 \| 2.2 \| 65.2 \| 0.9 \| 1.4 \| \| General surgery \| BPH(open prostatectomy) \| 20 \| 2 \| 45 \| 4 \| 11.3 \| 10 \| 0.2 \| GSH \| \| Thoraco-abdominal trauma (emergency Laparotomy) \| 11 \| 8 \| 28 \| 24 \| 1.2 \| 72 \| 2.2 \| 3.2 \| \| Elective laparotomy \| 5 \| 0 \| 5 \| 0 \| NA \| 0 \| 0 \| GSH \| \| Subdural/epidural hematoma (bur hole) \| 10 \| 2 \| 15 \| 5 \| 3 \| 20 \| 0.5 \| GSH \| \| Goiter (thyroidectomy) \| 30 \| 3 \| 42 \| 6 \| 7 \| 10 \| 0.2 \| GSH \| \| Breast Ca (Mastectomy) \| 14 \| 3 \| 28 \| 5 \| 5.6 \| 21 \| 0.4 \| GSH \| \| Cholelithiasis (cholecystectomy) \| 0 \| 0 \| 0 \| 0 \| NA \| NA \| NA \| GSH \| \| Appendicitis(appendectomy) \| 0 \| 0 \| 0 \| 0 \| NA \| NA \| NA \| GSH \| \| Pediatrics surgery(elective and emergency) \| 6 \| 2 \| 6 \| 2 \| 3 \| 33 \| 0.3 \| GSH \| \| Others \| 7 \| 2 \| 10 \| 2 \| 5 \| 28 \| 0.3 \| GSH \| \| Orthopedic procedures \| Elective \| 24 \| 16 \| 44 \| 33 \| 1.3 \| 66 \| 1.4 \| 2.1 \| \| Emergency \| 21 \| 18 \| 50 \| 42 \| 1.2 \| 85 \| 2 \| 3 \| \| Total \| \| 412 \| 138 \| 773 \| 278 \|  \|  \|  \|  \|   **NB-** C/T(Cross-match to transfusion ratio); %T(Transfusion probability); TI(Transfusion index); MSBOS(Maximal Surgical Blood Order Schedule).  Depending on the urgency of the procedures majority 507(65.6%) of cross-matched units were done in elective procedures (**Figure 1**).  Figure 1: Over all utilization of cross-matched blood in Debre Markos Comprehensive Specialized Hospital from September, to February 2021/2022 (N=1008). | 5-10 |
| DISCUSSION | Developing a blood ordering policy, which is a guide to expect normal blood usage for surgical procedures, can decrease the over-ordering of blood thereby reducing unnecessary compatibility testing, and returning unused blood. The maximum Surgical Blood Order Schedule (MSBOS) is used as guidelines for frequently performed surgical procedures by recommending the maximum number of units of blood to be cross-matched preoperatively (16, 17). In the absence of MSBOS, patients are exposed to unnecessary additional costs of health care. Chawla, T. et. al. reported that there is a possibility of direct savings of health care costs by 90.87% to 23.33% if MSBOS were applied in surgical patients(9). In current study 773 blood units were cross-matched out of the ordered units, only 278(35.9%) units of blood were transfused for 138 patients. This indicates that 64.1% of units of blood were unutilized. Depending on the urgency of the procedures majority 507(65.6%) of cross-matched units were done in elective procedures (**Figure 1**), this results in unnecessary health care burden on health professionals and cost on patients.  One of the challenges in developing MSBOS is anesthetists’ and surgeons’ readiness to accept it. The hospital blood bank’s ability to supply blood and blood components rapidly in times of emergency affects the professionals’ confidence to accept MSBOS(16). In this study, the overall transfusion rate for all surgeries was 35.9%,which is better trend tthan a study, done by Aryal S, et al (2016) they targeted formulating a maximum surgical blood ordering schedule in Obstetrics and Gynecology; they found that the overall transfusion rate for all surgeries was 22.5%(7). The cause for variation in blood utilization might be in their study the type of procedures included in the study are gynecological and obstetrics surgeries only.  In this study, among all cross-matched units in both elective and emergency surgeries, 64.1% of blood units were left unutilized. On the other hand, in elective procedures, the unutilized rate was higher (77.7%). In emergency procedures majority of cross-matched units were transfused means 64.3% of units utilized ,which is similar with a study done by CHALYA.P.L, et al.who assess blood transfusion practice found a large number of cross-matched units left unutilized (71.8%) (2). But in emergency surgeries blood utilization is much better in our study area than CHALYA.P.L, et al’s (64.1 vs 26.9%)  Regarding, overall blood transfusion indices we found significant values in both %T and TI 33.5%, and 0.7 respectively. But the C/T (2.9) ratio was not significant. A study done by Yangon, et al. on Blood requisition versus utilization practice found that the overall C/ T ratio, %T, and TI were 1, 73.07%, and 1.49 respectively (19). Their study showed better utilization indices, the reason might be they included non-surgical patients, for whom requisition is made only when 100% is necessary/indicated for transfusion. Better usage was also observed in terms of blood utilization indices with the value of C/T ratio of 5.95, %T of 67.7%, and TI = 0.87 respectively(20). Rehan M. et al, found blood utilization approaching the ideal which was overall C/T was1.3, %T 76.7%, TI 0.9(21). In the other study done by Yazdi A.P. et al., the utilization rate was insignificant in their study area; The transfusion indices were as follows C/T ratio, %T, and TI were 3.71, 16.83%, and 0.31, respectively (22).  In this study,the overall blood transfusion indices for elective and emergency surgery sets respectively as follows C/ T ratio (4.6 vs 1.5), %T (22 vs 78.8), and TI(0.4 vs 1.9). Our study is also consistent with a study done by CHALYA.P.L, et al. The overall C/T ratio, %T, and TI in the elective operations were 5.8, 15.9%, and 0.2 respectively (2). In the other study done by Haghpanah S, et al. The overall C/T ratio, T%, and TI were 2.49, 46.6%, and 0.83 respectively for all elective procedures (23). This showed better utilization of blood than the current study setup. The reason might be in their study set up surgical procedures are more critical like cardiac, thoracic, and kidney surgeries. Blood transfusion indices (C/T, T%, and TI) be affected by many factors like differences in surgical and anesthetic techniques, preoperative conditions of the patients, and variations in transfusion protocols (24, 25).  In this study, there is blood utilization indices variation among different types of surgical procedures. Among elective procedures, blood utilization was significant in orthopedic surgery with the value of C/T ratio 1.3, %T 66, and TI 1.4. In the rest of the elective procedures, blood transfusion indices were not significant as the C/T ratio, %T, and TI values mentioned in (table 3). Among emergency procedures, blood utilization indices are not significant in epidural/subdural hematoma /bur hole. On the other hand, blood utilization is highest in uterine rupture (emergency hysterectomy) with the value of C/T ratio 1.2, %T 100, and TI 4.3. Similarly, with our study, Mauka W.L.et al. also found that the highest risk factor for blood transfusion was being in the orthopedic ward (RR 3.8; 95% CI 2.2, 6.7) compared with other wards; in a general surgical ward (RR 3.3; 95% CI 2.7, 4.2), pediatric ward (RR 1.8; 95% CI 1.2, 2.7), obstetric ward (RR 2.5; 95% CI 2.0, 3.1), gynecological ward (RR 2.1; 95% CI 1.5, 2.9) (26). | 11-12 |
| Conclusion | The overall blood utilization indices in emergency surgical patients were better than in elective and preoperative grouping, screening, and hold (GSH) is sufficient for elective surgical procedures to decrease wastage of valuable supplies. | 12 |
| Conflict of interest | the authors declare that there is no conflict of interest | 12 |
| Funding | The authors received no financial support for the research authorship/or publication of this article | |
| Contributor ship | All authors made a significant contribution to the work reported, whether that is in the conception, study design, execution, acquisition of data, analysis, and interpretation, or in all these areas; took part in drafting, revising, or critically reviewing the article. | |
| Acknowledgment | Our special thanks go to Debre Markos Specialized Hospital staff, for their assistance and cooperation in the completion of this study. We thank also all the study participants for their voluntary participation during data collection and also wish to thank data collectors and supervisors. | |
